# Comparative Evaluation of Rice Bran Wax as an Ointment Base with Standard Base

**DOI:** 10.4103/0250-474X.51965

**Published:** 2009

**Authors:** Vidya Sabale, P. M. Sabale, C. L. Lakhotiya

**Affiliations:** Parul Arogya Seva Mandal's Baroda College of Pharmacy, Limda, Vadodara-391 760, India; 1 Pharmacy Department, Faculty of Technology and Engineering, The M. S. University of Baroda, Vadodara-390 001, India; 2 Department of Pharmaceutical Sciences, R. T. M. Nagpur University, Nagpur-440 010, India

**Keywords:** Rice bran wax, ointment base, spreadability test, water number

## Abstract

Waxes have been used in many cosmetic preparations and pharmaceuticals as formulation aids. Rice bran wax is a byproduct of rice bran oil industry. Present investigation has been aimed to explore the possible utility of rice bran wax as ointment base compared to standard base. The rice bran wax obtained, purified and its physicochemical characteristics were determined. Ointment base acts as a carrier for medicaments. The ointment base composition determines not only the extent of penetration but also controls the transfer of medicaments from the base to the body tissues. Rice bran wax base was compared with standard base for appearance, spreadability, water number, wash ability and diffusibility. The results show that rice bran wax acts as an ointment base as far as its pharmaceutical properties are concerned and it could effectively replace comparatively costlier available ointment bases.

Waxes (animal and plants) are esters of high molecular weight monohydroxy alcohols and high molecular weight carboxylic acids. They are chemically different from fats and oils, from hydrocarbon or paraffin waxes, and from synthetic polyether waxes such as carbowax[1]. Recent work indicates that rice bran wax is mainly an ester of lignoceric acid and myricyl alcohol. The hard non-tacky wax separately recovered is reported to be chiefly melissyl cerotate and melts at about 80-85°. Research at Southern Regional Research Laboratories (SRRL) showed that the properties of the refined and bleached wax are similar to those of presently imported carnauba wax[2].

Ointments are used topically for several purposes like protective, antiseptic, emollient, antipruritic, keratolytic and astringent. The base of an ointment is of prime importance if the finished product is expected to function as any of the above categories. Ointment base acts as a carrier for medicaments. The ointment base composition determines not only the extent of penetration but also controls the transfer of medicaments from the base to the body tissues[3,4].

Many waxes such as white wax, carnauba wax, etc have been tried for cosmetic formulations and have been used as pharmaceutical aids. Compared to these waxes rice bran wax is cheap and obtained from natural source (*Oryza sativa*, Family Graminae) and is abundantly available. It is an important byproduct of rice bran oil industry. Therefore, the present study was carried out to compare its properties and its utility as ointment base. It was the main objective of the present study to investigate whether any ointment base characteristics are associated with rice bran wax or not. It is also hoped that present investigation would provide additional data and information to the cosmetic chemists.

Abbe's refractometer, Soxhlet extractor, desiccator and spreadability test apparatus were employed for the present study. All other chemicals and solvents used were of analytical grade. Crude rice bran wax was obtained from solvent extraction plant, Gondia, Maharashtra, and it was purified and standardized and used[5–7]. A mixture of 5% white wax and 95% white petrolatum ointment base was prepared which is used as standard base for comparison with rice bran wax base which was prepared by incorporating 5% rice bran wax and 95% white petrolatum. Rice bran wax base was compared with standard base for appearance, spreadability, water number and diffusibility. Washability was determined by rubbing the little amount of base on the hand and washed off with warm water without using soap.

Ointment base should spread easily without too much drag and should not produce greater friction in the rubbing process. Spreadability was calculated by spreadability apparatus made of wooden board with scale and two glass slides having two pans on the both sides mounted on a pulley. An excess of sample was placed between two glass slides and 1000 g weight was placed on glass slide for 5 min to compress the sample to uniform thickness. Weight (250 g) was added to the pan. The time in sec. required to separate two slides was taken as a measure of spreadability[8]. S = m.L/t, where m is the weight tied on upper slide; L is the length of glass slide moved and t is the time in sec.

Water number is the maximum amount of water that can be added to 100 g of base at a given temperature. It was determined by continuously stirring the base with the addition of distilled water. When no more water was absorbed into the base evidenced by droplets of water remaining in the container was taken as end point[9].

Diffusibility gives the amount of ointment diffused with the body surface. For this salicylic acid ointment was prepared by using standard base and rice bran wax base (salicylic acid 2 g and ointment base 98 g). Nutrient agar medium was prepared using beef extract 10 g, peptone 10 g, sodium chloride 5 g, agar 1.2 g and distilled water 1000 ml. This was poured into Petri dish, hole was made in the centre of the medium and the ointment was then applied to the hole and time for diffusion was noted evidenced by pink rings on agar medium after particular interval[9–11].

Rice bran wax base appeared shining yellowish white with less wash ability as that of shining white colored standard base. The results show that rice bran wax base has good spreadability (average spreadability 16.8±2.4) as that of standard base (average spreadability 10.13±1.66). Ointment prepared with rice bran wax base showed good diffusibility (average diffusibility 0.6±0.057) than the ointment which was prepared using standard base (average diffusibility 0.4±0.057) and also showed decrease in water number as compared to standard base which represents its less water absorbing capacity (1.5 ml vs 2.0 ml for standard base). Thus results show that, rice bran wax acts as an oleaginous ointment base as far as its pharmaceutical properties are concerned against the available costly bases.
